# Seismic Performance of F3D Free-Form Structures Using Small-Scale Shaking Table Tests

**DOI:** 10.3390/ma15082868

**Published:** 2022-04-14

**Authors:** Min Jae Park, Gain Cheon, Robel Wondimu Alemayehu, Young K. Ju

**Affiliations:** School of Civil, Environmental and Architectural Engineering, Korea University, Seoul 02841, Korea; alswo8739@korea.ac.kr (M.J.P.); dooin1024@korea.ac.kr (G.C.); robel@korea.ac.kr (R.W.A.)

**Keywords:** F3D, free-form structures, shaking table test, small-scale, seismic performance

## Abstract

In recent years, studies that can maximize irregularity have increased as technological constraints weaken owing to the development of construction technology and the increase in demand for free-form structures. Considering this, free-form structures have been constructed using various materials. Concrete is considered most suitable for realizing an atypical shape because it is highly economical and can be assembled in a free form. However, not many studies have evaluated the structural performance of free-form concrete structures using free-form formwork 3D printer (F3D) technology, a 3D printing technology. Free-form structures must be designed to secure structural stability under both dead and live loads, as well as natural hazards such as wind, snow, and earthquakes. Therefore, in this study, we tested a free-form structure constructed by F3D printing using small-scale models that satisfy the similitude law with shaking tables. Furthermore, a finite element analysis was conducted to validate the small-scale tests. Lastly, the seismic performance of free-form concrete structures was evaluated based on the test and analysis results.

## 1. Introduction

**Figure 1:**
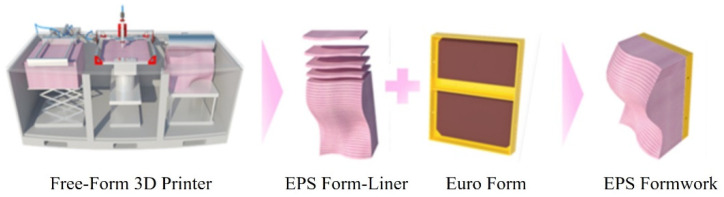
Process of EPS formwork.

Novel free-form structures have been actively designed in the modern construction field owing to the development of 3D printers. Free-form structures, as landmarks of cities or countries, have largely influenced the social, cultural, and economic aspects of the societies. In Korea, Tri-bowl and Dongdaemun Design Plaza, representative free-form structures, have been recently constructed because of the revision of landscape law in Korea that improved the landscape view in terms of beauty for infrastructures and buildings. Thus, the demands for free-form structures are increased, and various studies on construction materials and manufacturing technologies improving free-form shapes were conducted [1,2,3,4,5,6,7,8]. Especially the concrete was paid attention for free-form structural materials because of its usability and economical [2,3,4,5]. To install the concrete with a free-form shape, the study on molds was also conducted [3]. In addition, 3D printing technologies using concrete were also developed due to the easy way to manufacture free-form shapes [4,7]. One of the 3D printing technology manufacturing molds for free-form structures is the Free-form Formwork 3D printer (F3D), as shown in Figure 1. The F3D is a kind of technology manufacturing expanded polystyrene (EPS) foam liner using 3D printer based on laminated object manufacturing (LOM). The plate-type EPS foam is cut by laser or heat rays and laminated to form the molds [9].

The free-form structures manufactured by 3D printers must resist not only dead and live loads but also wind, snow, and earthquake loads to become stable structures [10,11]. In particular, all structures, including free-form structures, must secure stability under earthquake loads because earthquakes can cause numerous casualties and loss of property. However, the studies on seismic performance of free-form structures were barely conducted, whereas the studies on the design, construction, and material of free-from structures have been actively performed [12,13,14]. In addition, various researches about seismic evaluation for irregular-shaped structural systems were already proposed, whereas research about the seismic evaluation for irregular-shaped structural elements was hardly conducted. There are two evaluation methods for seismic performance, numerical and experimental approaches. In the case of experimental approaches, it is quite difficult to conduct full-scale tests because of the limitations of the shaking tables. Therefore, small-scale tests with small-scale shaking tables were generally conducted with similitude laws.

To evaluate the seismic performance of the structures, several indicators should be investigated [15,16,17,18]. One of the significant indicators is the story drift of the structures. Sommer and Bachmann [19] selected a story drift as one of the indicators for studying the seismic behavior of asymmetric reinforced concrete wall buildings. In addition, Rahman et al. [20] also used a story drift as one of the indicators for evaluating the seismic performance of reinforced concrete buildings by Bangladesh, India, and United States codes. Hu et al. [21] investigated the seismic performance and behavior of composite-moment frames based on the inter-story drift ratio, peak response at the roof, and stability coefficient defined by the deflection amplification factor. Zou et al. [22] decided a story drift as a seismic performance criterion for base-isolated concrete buildings. The design optimization procedure was proposed to minimize the total cost using reliability analysis. Carrillo and Alcocer [23] conducted a performance-based seismic design of reinforced concrete walls for low-rise housing based on the allowable story drift ratios considering crack width according to performance levels. The seismic performance was evaluated by story drifts obtained from experimental studies. Özhendekci and Özhendekci [24] performed inelastic dynamic analyses using the OPENSEES program for seismic performance of 10-story steel special moment-resisting frames. In this study, a story drift was chosen to evaluate the seismic performance. Abou-Elfath et al. [25] investigated the seismic performance of moment-resisting steel frames by story drift criteria according to Egyptian code.

This paper aims to investigate the seismic performance of F3D free-form structures using small-scale shaking tables. To validate the small-scale tests, the similitude laws were used for designing the SDOF model and small-scale model from full-scale F3D free-form structures. From the free vibration tests, the natural frequencies of SDOF and small-scale models were obtained and the seismic behavior of SDOF and small-scale models were investigated under sinusoidal and earthquake excitations. In addition, the response spectrum method [26], the most common method in seismic design, was selected to obtain the dynamic response of full-scale free-form structures via finite element analysis. Using the response spectrum method, not only the time history curve for displacements but also peak displacement of the structures under earthquake can be easily obtained. The tests results were compared to finite element analysis results to obtain validation of small-scale shaking table tests and evaluate the seismic performance of F3D free-form structures using story drift limits.

## 2. F3D Free-From Structures

### 2.1. Overview

**Figure 2:**
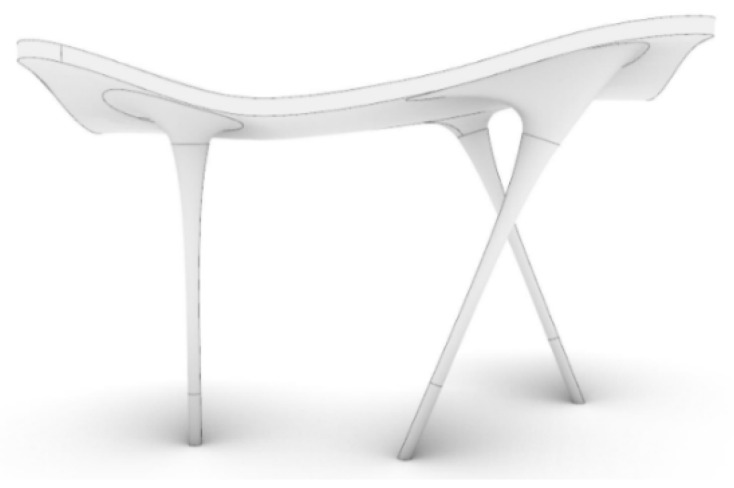
Concept of free-form structure.

The F3D free-form structure in this study is a kind of free-form concrete structure of which size is 3.6 m (length) × 3.0 m (width) × 2.5 m (height), imitating the Meiso no Mori municipal federal hall in Japan [27]. The structure has a streamlined shape roof and curvature columns as shown in Figure 2. This free-form structure was planned to be manufactured by the ultra-high performance concrete (UHPC) from F3D 3D printers and its design showed an irregular-shaped of structures. In addition, UHPC, of which compressive strength is 180 MPa, was selected as the material because UHPC contributed to a slender section for making irregular-shaped [28].

### 2.2. Manufacturing Process

**Figure 3:**
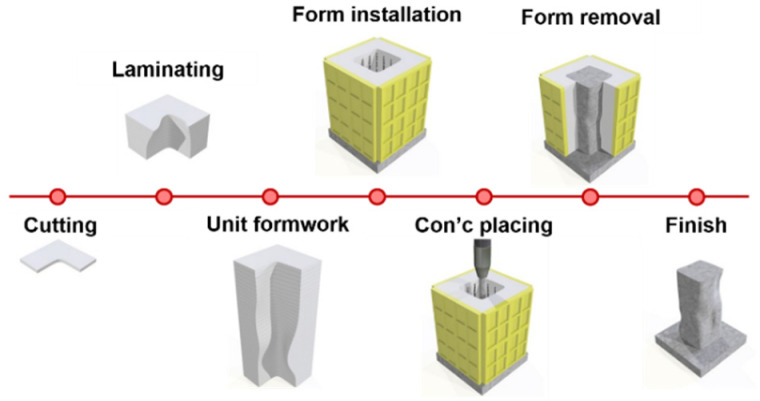
Manufacturing process of free-form concrete structure with EPS foam molds.

To rapidly and economically manufacture the free-form concrete structure, the EPS foam was used for mold owing to its lightweight, low cost, and easy fabrication. The F3D 3D printing technique was applied to manufacture the EPS foam molds for free-form concrete structure based on the LOM, which is available for rapid production speed and enough precision for a large element like EPS foam. In this study, EPS foam was selected owing to its significant features instead of steel molds, which is a typical form for free-form structures. The manufacturing process for the free-form concrete structure with EPS foam molds fabricated by LOM-based 3D printers is shown in Figure 3 [29]. The first step is to cut the EPS foams according to each layer plan. The second step is to stack the cut EPS foams to form the molds. The third step is to install the exterior form for resisting later pressure from concrete curing. The fourth step is to place concretes into the molds and the final step is to cure them and remove the form and the EPS foam molds when the compressive strength of concrete reaches 5 MPa [30].

## 3. Small-Scale Shaking Table Tests

### 3.1. Overview

To evaluate the seismic performance of free-form structures, the shaking table tests were conducted. The height, natural period, and natural frequency of a full-scale free-form structure are described in Table 1.

**Figure 4:**
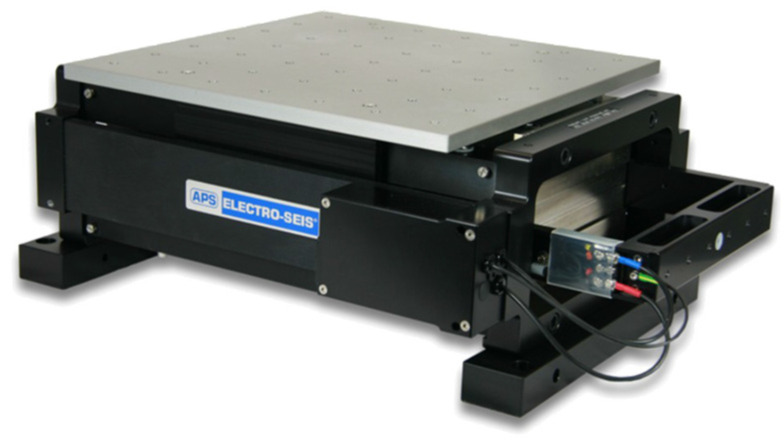
Uniaxial small-scale shaking table.

Due to the limitation of the shaking table, the uniaxial small-scale shaking table tests with small-scale free-form structures were conducted. The specification of the uniaxial small-scale shaking table is presented in Figure 4 and Table 2. The shaking table can apply the forced sinusoidal, random, and earthquake wave and the accelerometer installed at the shaking table plate can measure and check its stability.

### 3.2. Similitude Law

Owing to the limitations of the small-scale shaking table, small-scale tests should be conducted. The similitude law should be considered to alleviate the difference between small-scale and full-scale tests. The reduction rates of structural parameters depending on the height ratio (*h = H_full-scale_*/*H_small-scale_*) by similitude law are presented in Table 3 [31].

### 3.3. Test Specimens

**Figure 5:**
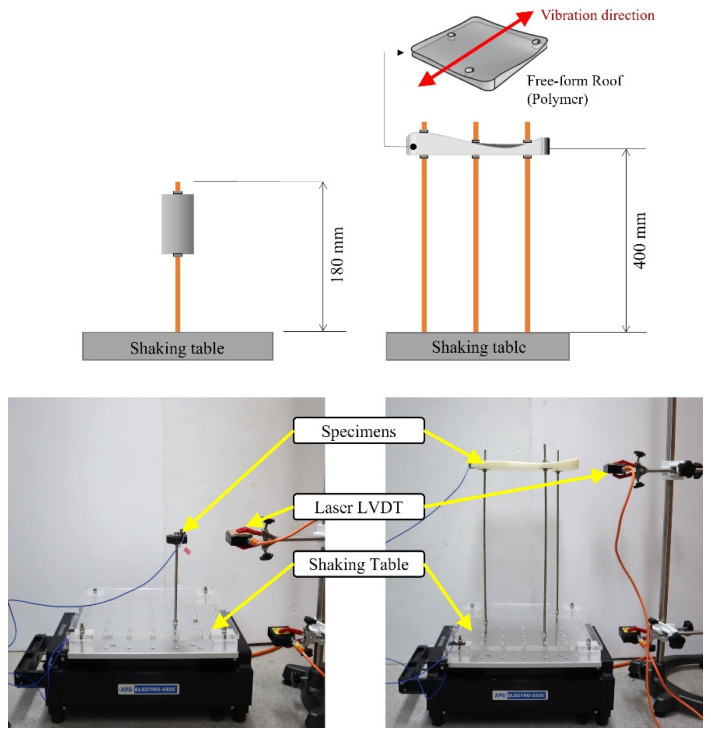
Test setup.

This study selected two test specimens, the SDOF model and the small-scale models. The SDOF model consists of a brass bar with a length of 180 mm and circular plates at the top of the brass bar for weight. The small-scale model consists of three brass bars with a length of 400 mm and a free-form roof fabricated by a 3D printer for weight. The test setup is shown in Figure 5. Cantilever support and both fixed ends support were modeled for the SDOF and the small-scale models, respectively. The Laser LVDT to measure the displacement at the top of the specimens was installed next to the shaking table. The weights of each model were decided by dynamic properties as described in Table 4 [32]. The direction of input vibrations was decided as the estimated weakest direction (Figure 5).

### 3.4. Applied Vibration Plan

**Figure 6:**
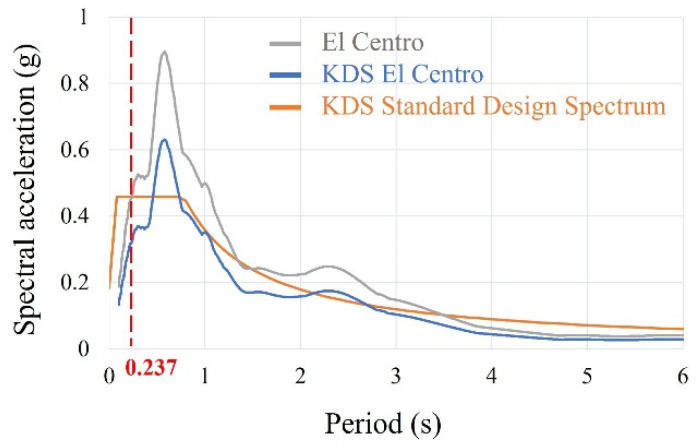
Earthquake vibration.

Three vibrations were applied to the test specimens, free vibration, sinusoidal forced vibration with constant amplitude and period, and earthquake vibration. For free vibration, the initial displacement (8 mm) was applied to the test specimens. For sinusoidal forced vibration, the vibration with various frequencies ranging approximately from 0.7*f* to 1.2*f* was applied with constant amplitude and period during 30 s. From the free vibration, the damping ratios of the test specimens were obtained and the natural frequencies were also obtained from the sinusoidal forced vibration with constant amplitude and period [33,34]. In addition, sinusoidal force vibration with a specific frequency and designed peak acceleration by an earthquake was applied to the small-scale specimens to measure the dynamic behavior compared to finite element analysis. For earthquake vibration, the El Centro earthquake was selected because it is quite similar to the Korean design standard (KDS) response spectrum and is widely used by Korean structural engineers. However, the earthquake should be scaled because Korea belongs to a low-to-moderate seismic zone. Therefore, the earthquake vibration was scaled based on the natural period (0.237 s) of a full-scale free-form structure (Table 1), as illustrated in Figure 6.

### 3.5. Test Results

#### 3.5.1. Free Vibration

**Figure 7:**
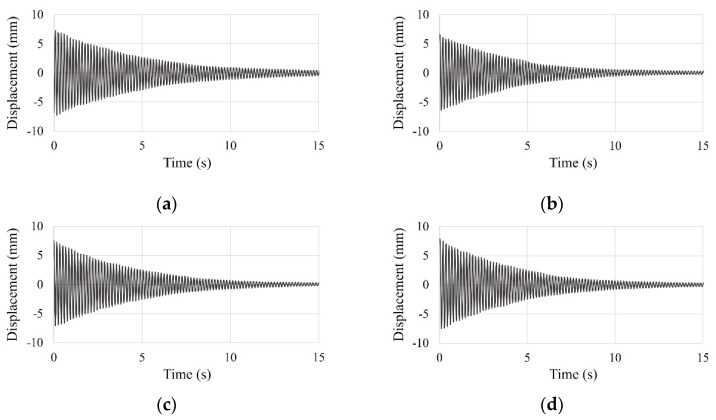

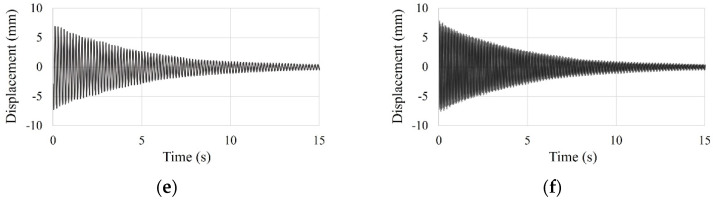
Time history curve of the SDOF model by free vibration test: (**a**) first test; (**b**) second test; (**c**) third test; (**d**) fourth test; (**e**) five test; (**f**) overlapping entire tests.

**Figure 8:**
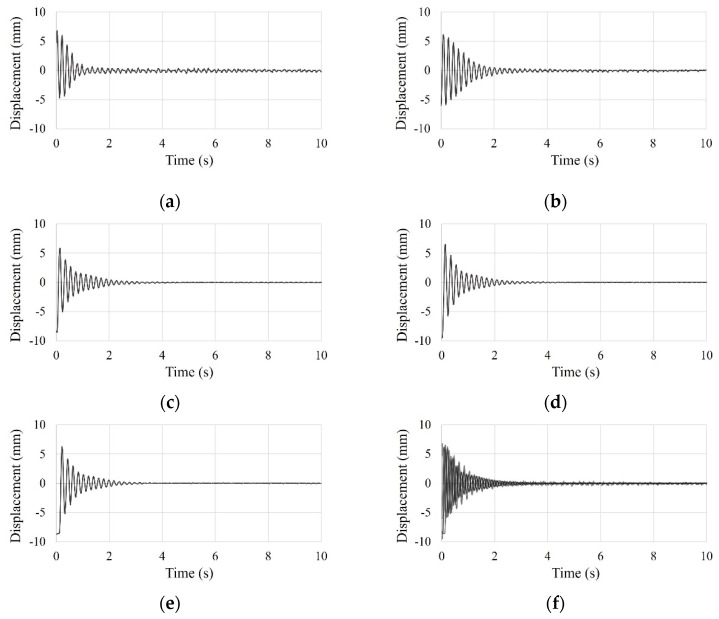
Time history curve of the small-scale model by free vibration test: (**a**) first test; (**b**) second test; (**c**) third test; (**d**) fourth test; (**e**) five test; (**f**) overlapping entire tests.

To improve the reliability of test results, the test was conducted five times. From the initial displacement (approximately 8 mm), the test data of the SDOF and the small-scale models was measured for 30 s. However, the only test results of the SDOF model for 15 s are shown in Figure 7, as test data after 15 s was negligible. And the only test results of the small-scale model for 10 s are shown in Figure 8, as test data after 10 s was negligible.

**Figure 9:**
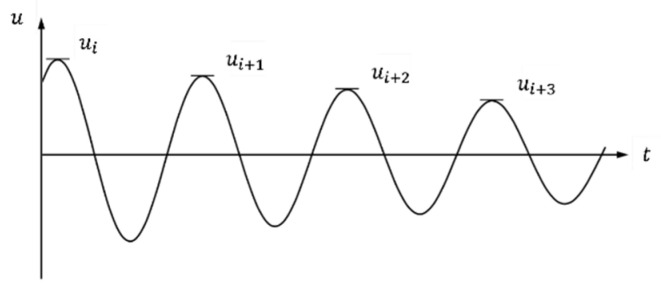
Example of time history curve of the free vibration.

The damping ratio can be obtained from the time history curve of the free vibration test using the logarithmic decrement method [33,35]. The logarithmic decrement method is to calculate the damping ratio (ξ) with reduction peak amplitude (*u*) for *j* cycles (Figure 9) under free vibration as follows:

(1) $$\xi =\frac{1}{2\pi }\left(\frac{1}{j}\ln\frac{u_{i}}{u_{i+j}}\right)$$

where *ξ* is the damping ratio, *i* and *j* is the number of cycles, *u_i_* is the peak amplitudes at the *i-th* cycles

Based on the method, damping ratios of the SDOF and small-scale models under free vibrations are presented in Table 5. The average damping ratios of the SDOF and the small-scale models were 0.90% and 3.30%, respectively.

#### 3.5.2. Sinusoidal Forced Vibration

**Figure 10:**
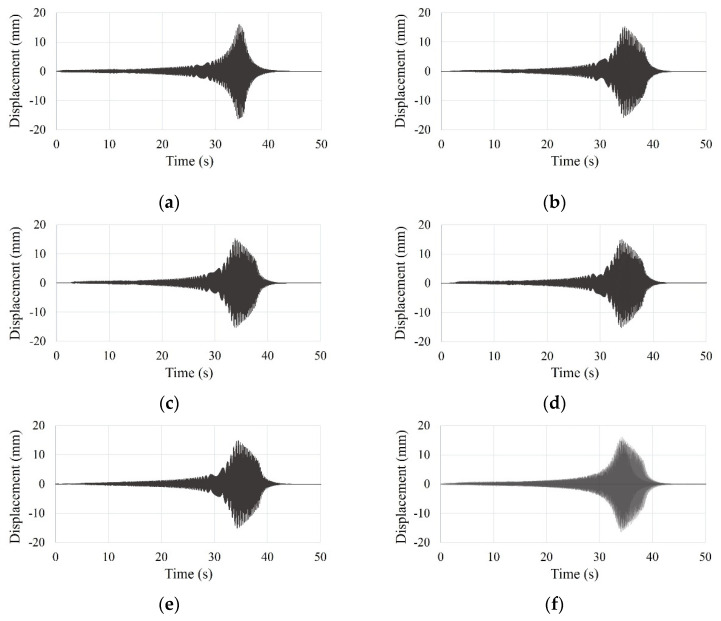
Time history curve of the SDOF model by sinusoidal forced vibration test: (**a**) first test; (**b**) second test; (**c**) third test; (**d**) fourth test; (**e**) five test; (**f**) overlapping entire tests.

**Figure 11:**
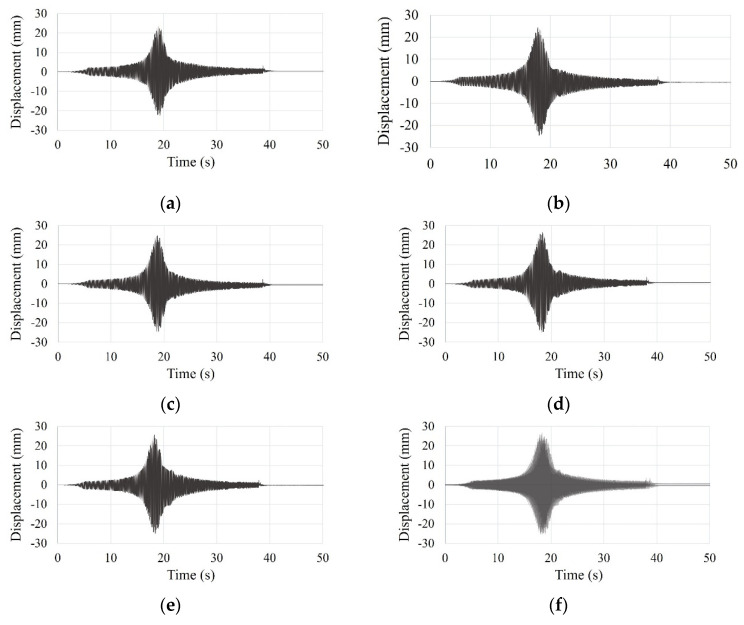
Time history curve of the small-scale model by sinusoidal forced vibration test: (**a**) first test; (**b**) second test; (**c**) third test; (**d**) fourth test; (**e**) five test; (**f**) overlapping entire tests.

The sinusoidal forced vibrations, increasing the frequencies from 0.7*f* to 1.2*f*, applied to test specimens with constant peak amplitudes. The peak amplitudes for the SDOF and the small-scale models were 16 and 25 mm, respectively. The time history curves of the SDOF and the small-scale models under sinusoidal forced vibration with varying frequencies are shown in Figure 10 and Figure 11.

From the sinusoidal forced vibration tests, the natural frequencies of the SDOF and small-scale models were obtained and compared to theoretical values (Table 6).

**Figure 12:**
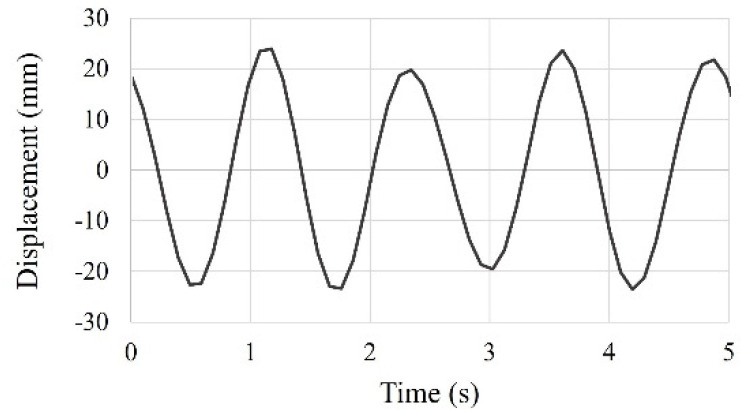
Time history curve of the small-scale model under sinusoidal forced vibration equal to scaled El Centro earthquake.

The sinusoidal forced vibration test with constant frequency and peak acceleration, equal to scaled El Centro earthquake wave, was also conducted to evaluate the seismic behavior of the small-scale model. The test result for only 5 s is shown in Figure 12 because the time history of this test showed repetitive behavior.

#### 3.5.3. Scaled El Centro Earthquake Vibration

**Figure 13:**
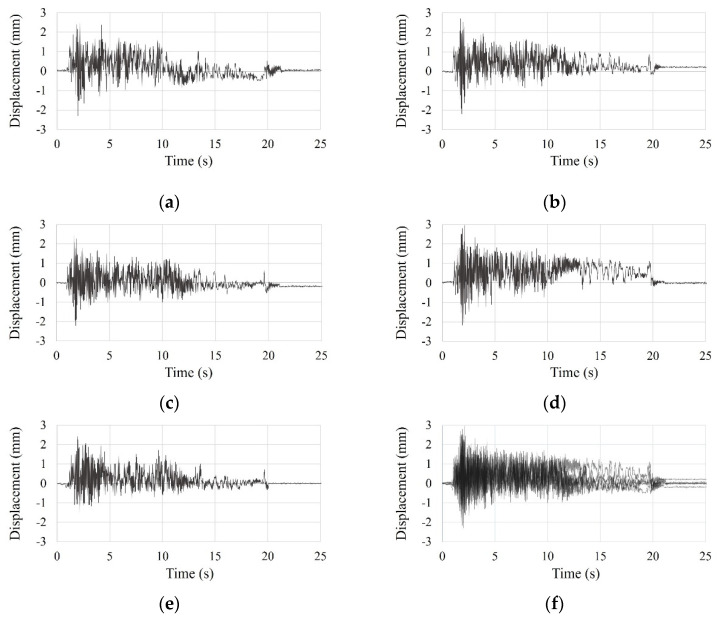
Time history curve of the SDOF model by scaled El Centro earthquake vibration test: (**a**) first test; (**b**) second test; (**c**) third test; (**d**) fourth test; (**e**) five test; (**f**) overlapping entire tests.

**Figure 14:**
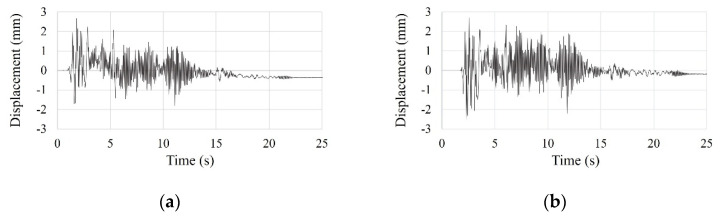

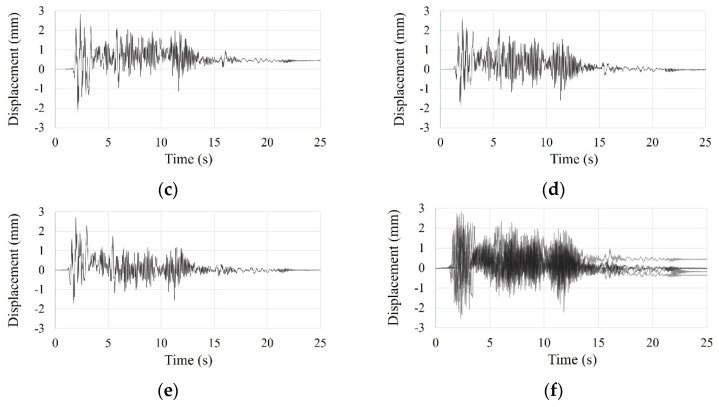
Time history curve of the small-scale model by scaled El Centro earthquake vibration test: (**a**) first test; (**b**) second test; (**c**) third test; (**d**) fourth test; (**e**) five test; (**f**) overlapping entire tests.

The time history curves of the SDOF and the small-scale models under scaled El Centro earthquake vibration are shown in Figure 13 and Figure 14.

From the scaled El Centro earthquake vibration tests, the maximum displacements of the SDOF and small-scale models were obtained (Table 7).

### 3.6. Finite Element Analysis

**Figure 15:**
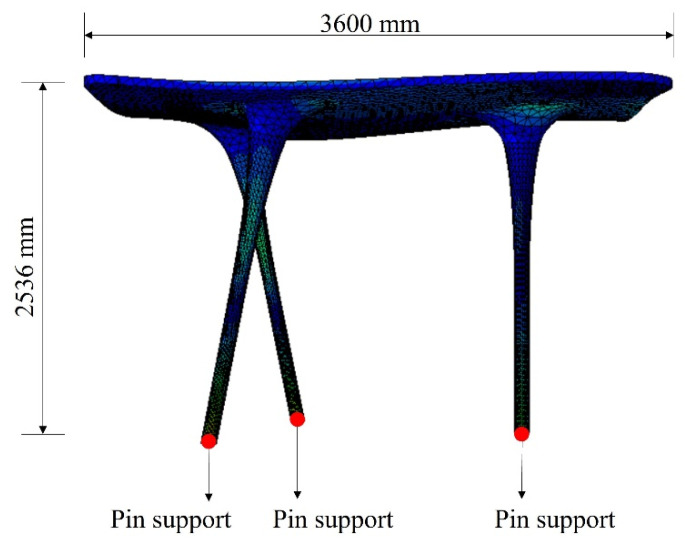
Finite element model.

The finite element analysis for full-scale free-form concrete structures under the sinusoidal forced vibration and scaled El Centro earthquake vibration was conducted. The Abaqus/CAE 2017 program was used for the analysis and an 8-node solid element was selected. Because the purpose of the analysis was only to validate the small-scale shaking table tests, the mesh size was 60 mm, which was verified by various numerical studies [36,37,38,39]. The UHPC material properties were modeled by the guideline [40] and the Drucker Prager Hardening material model [41], as presented in Table 8. The damping ratio of UHPC material was assumed to be 5%, which is a commonly used value for reinforced concrete [42]. First, the frequency analysis was conducted to calculate the natural frequency. The obtained natural frequency of the full-scale free-form structure via the analysis was 4.22 Hz. In addition, the 2nd and 3rd mode frequencies were 5.00 Hz and 6.71 Hz, respectively. The configuration of the full-scale free-form concrete structure is shown in Figure 15. The dynamic, implicit analysis was conducted to obtain the dynamic responses that were time histories of displacement of full-scale free-form structures.

**Figure 16:**
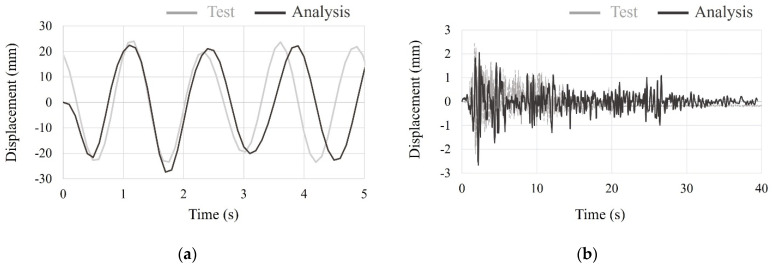
Analysis and test results of the small-scale model by: (**a**) sinusoidal forced vibration; (**b**) scaled El Centro earthquake vibration.

To compare the time history of full-scale free-form concrete structures with the small-scale shaking table test, the sinusoidal forced vibration, which is equal to scaled El Centro earthquake vibration, and scaled El Centro earthquake vibration was applied. The analysis results compared with the test results are shown in Figure 16. As the analysis results of the small-scale model by both sinusoidal forced vibration and scaled El Centro earthquake vibration were quite similar to test results validated by the similitude law, small-scale free-form structure by small-scale shaking tests showed reliable results.

### 3.7. Seismic Performance

The allowable story drift limit is one of the main factors to evaluate the seismic performance of structures when the structures are intact under earthquakes [19,20,21,22,23,24,25]. In Korea, the allowable story drift is *h_sx_*/100 for the most important buildings [43] and Federal Emergency Management (FEMA) limited story drift for the highest performance as *h_sx_*/100 for the concrete frames [44]. The allowable story drift limit of a concrete frame in the Eurocode 8 was proposed as *h_sx_*/200 for buildings having non-structural elements of brittle materials attached to the structure [45]. The largest story drift under scaled El Centro earthquake vibration among the test and analysis results was the finite element analysis result (2.66 mm), which was approximately *h_sx_*/1000. Therefore, the introduced full-scale free-form UPHC structure had enough seismic performance under scaled El Centro earthquake evaluated by the small-scale shaking table tests and finite element analysis.

## 4. Conclusions

This paper aims to evaluate the seismic performance of the free-form concrete structures fabricated by F3D printing technology. The dynamic properties of the SDOF and small-scale models were investigated using a uniaxial shaking table with the similitude law. The tests results were compared with results of finite element analysis that conducted a dynamic analysis of the full-scale free-form concrete structure under scaled El Centro earthquake vibration to enhance the validation of small-scale shaking table tests. The findings of this paper may be summarized as follows:

(1) The introduced small-scale shaking table tests were validated by the comparison between theoretical and experimental frequencies. The theoretical frequency was obtained from the similitude law considering the height ratio (full-scale structures/small-scale structures). The experimental frequencies were obtained from the small-scale shaking table tests with the sinusoidal forced vibration.
(2) The small-scale shaking table test results had a close agreement with the finite element analysis results of full-scale free-form structures under the scaled El Centro earthquake. With this investigation, the small-scale shaking table tests were sufficiently validated to study the seismic behavior of full-scale free-form structures.
(3) As the difference between analysis and test results under sinusoidal forced and scaled El Centro earthquake vibration was small, evaluating seismic performance using small-scale shaking table tests is a reliable way for free-form concrete structures.
(4) The maximum story drift of free-form concrete structures fabricated by F3D printing technology obtained from the tests and analysis was approximately 0.1% of story height, which was quite lower than the allowable story drift in several countries.

## Figures and Tables

**Table 1:** Information of a full-scale free-form structure.

Height (*H*) (m)	Natural Period (*T*) (s)	Natural Frequency (*f*) (Hz)
2.54	0.237	4.22

**Table 2:** Specifications of a small-scale shaking table.

Parameter	Specifications
Name	APS400
Force (sine peak)	445 N
Velocity (sine peak)	1000 mm/s
Stroke (sine peak)	158 mm
Frequency range	200 Hz
Operation	Horizontal
Total shaker weight	86 kg
Overall dimension (length × width × height)	526 × 314 × 178 mm
Maximum test load weight	23 kg

**Table 3:** Reduction rates of parameters depending on the height ratio (*h*) by similitude law.

Parameters	Height	Time	Mass	Force	Acceleration	Stress	Strain
Height ratio (*h*)	1/*h*	1/(*h*^1/2^)	1/(*h*^2^)	1/(*h*^2^)	1	1	1

**Table 4:** Dynamic properties of test specimens.

Specimen	*H* (m)	*h*	*T* (s)	*f* (Hz)	Stiffness (*K_s_*) (N/m)	Mass (*M_s_*) (kg)
SDOF model	0.18	14.09	0.06314	15.84	1404	0.1418
Small-scale model	0.40	6.340	0.09413	10.62	1536	0.3446

**Table 5:** Damping ratio.

Test	SDOF Model	Small-Scale Model
1	1.01%	3.21%
2	0.87%	3.14%
3	0.92%	3.11%
4	0.95%	3.43%
5	0.75%	3.61%
Average	0.90%	3.30%

**Table 6:** Natural frequency.

Specimen	Natural Frequency (Hz)		Error (%)
	Theoretical Value	Experimental Value	
SDOF model	15.84	16.51	4.23
Small-scale model	10.62	10.24	3.58

**Table 7:** Maximum displacement.

Test	SDOF Model	Small-Scale Model
1	2.61 mm	2.51 mm
2	2.60 mm	2.38 mm
3	2.67 mm	2.49 mm
4	2.61 mm	2.55 mm
5	2.74 mm	2.52 mm
Average	2.65 mm	2.49 mm

**Table 8:** UHPC material properties.

Property	Value
Density	2190 kg/m^3^
Compressive strength	180 MPa
Tensile strength	14.6 MPa
Modulus of elasticity	45.0 GPa
Flexural strength	35.6 MPa
Poisson’s ratio	0.2

## Data Availability

Not applicable.
